# An Indirect Pathway from the Rat Interstitial Nucleus of Cajal to the Vestibulocerebellum Involved in Vertical Gaze Holding

**DOI:** 10.1523/ENEURO.0294-24.2024

**Published:** 2024-11-05

**Authors:** Taketoshi Sugimura, Toshio Miyashita, Mariko Yamamoto, Kenta Kobayashi, Yumiko Yoshimura, Yasuhiko Saito

**Affiliations:** ^1^Department of Neurophysiology, Nara Medical University, Kashihara 634-8521, Japan; ^2^Division of Visual Information Processing, National Institute for Physiological Sciences and School of Life Science, The Graduate University for Advanced Studies (SOKENDAI), Okazaki 444-8585, Japan; ^3^Department of Anatomy, Teikyo University School of Medicine, Itabashi 173-8605, Japan; ^4^Section of Viral Vector Development, National Institute for Physiological Sciences and School of Life Science, The Graduate University for Advanced Studies (SOKENDAI), Okazaki 444-8585, Japan

**Keywords:** eye movement, flocculus, interstitial nucleus of Cajal, medial vestibular nucleus, prepositus hypoglossi nucleus, rabies virus

## Abstract

The neural network, including the interstitial nucleus of Cajal (INC), functions as an oculomotor neural integrator involved in the control of vertical gaze holding. Impairment of the vestibulocerebellum (VC), including the flocculus (FL), has been shown to affect vertical gaze holding, indicating that the INC cooperates with the VC in controlling this function. However, a network between the INC and VC has not been identified. In this study, we aimed to obtain anatomical evidence of a neural pathway from the INC to the VC (the INC-VC pathway) in rats. Injection of dextran-conjugated Alexa Fluor 488 or adeno-associated virus 2-retro (AAV2retro) expressing GFP into the FL or another VC region (uvula/nodulus) did not reveal any retrogradely labeled neurons in the INC, suggesting that INC neurons do not project directly to the VC. Rabies virus-based transsynaptic tracing experiments revealed that the INC-VC pathway is mediated via synaptic connections with the prepositus hypoglossi nucleus (PHN) and medial vestibular nucleus (MVN). The INC neurons in the INC-VC pathway were mainly localized bilaterally within the rostral region of the INC. Transsynaptic tracing experiments involving the INC-FL pathway revealed that INC neurons connected to the FL via the bilateral PHN and MVN. These results indicate that the INC-VC pathway is not a direct pathway but is mediated via the PHN and MVN. These findings can provide clues for understanding the network mechanisms responsible for vertical gaze holding.

## Significance Statement

Gaze holding is crucial for achieving clear vision. However, the mechanism underlying vertical gaze holding is not fully understood owing to our limited knowledge of the neural connections between the interstitial nucleus of Cajal (INC) and the vestibulocerebellum (VC). In this study, we aimed to identify the neural pathway from the INC to the VC. Retrograde tract-tracing experiments using tracers and viruses revealed that INC neurons do not directly project to the VC; rather, the pathway from the INC to the VC is mediated via synaptic connections with the PHN and MVN. This finding clarifies a new indirect pathway from the INC to the VC that is responsible for vertical gaze holding.

## Introduction

During gaze holding, tonic contraction of the extraocular muscles is required to maintain eccentric eye positions against forces that pull the eyes back to the central position. Sustained neural activity for this tonic contraction is produced through the oculomotor neural integrator, which converts transient burst signals proportional to eye or head velocity into sustained signals proportional to eye position (Skavenski and Robinson, 1973; Robinson, 1975, 1989; Fukushima, 1987, 1991; Fukushima et al., 1992; Fukushima and Kaneko, 1995; Moschovakis, 1997; Leigh and Zee, 2015). The prepositus hypoglossi nucleus (PHN) and the interstitial nucleus of Cajal (INC) are involved in the integrators responsible for horizontal and vertical gaze holdings, respectively (Fukushima, 1987, 1991; Fukushima et al., 1992; Fukushima and Kaneko, 1995; Moschovakis, 1997; Leigh and Zee, 2015).

Previous studies have indicated that the neural mechanisms underlying signal transformation in neural integrators involve the vestibulocerebellum (VC) network (Robinson, 1974; Zee et al., 1981; Godaux and Vanderkelen, 1984; Kheradmand and Zee, 2011; Leigh and Zee, 2015; Beh et al., 2017; Shadmehr, 2017; Shemesh and Zee, 2019) as well as excitatory networks within integrators (Saito and Yanagawa, 2010; Leigh and Zee, 2015; Saito et al., 2017; Saito and Sugimura, 2020) and cholinergic and monoaminergic modulations (Navarro-López et al., 2004, 2005; Zhang et al., 2016, 2018; Saito and Sugimura, 2023). It is difficult for the PHN alone or the INC alone to produce a sufficient time constant for achieving gaze holding; rather, the neural networks, including these neural nuclei and the VC, may extend this time constant (Robinson, 1974; Kamath and Keller, 1976; Zee et al., 1980, 1981; Kheradmand and Zee, 2011; Leigh and Zee, 2015). Indeed, cerebellectomy or a local lesion within the VC, including in the cerebellar flocculus (FL), results in impaired horizontal and vertical gaze holding (Robinson, 1974; Zee et al., 1981; Godaux and Vanderkelen, 1984), causing the eyes to drift back toward the primary position. Therefore, it is important to identify the neural networks both between the PHN and VC and between the INC and VC to elucidate the mechanisms by which gaze holding is controlled (Zee et al., 1980, 1981; Kheradmand and Zee, 2011; Leigh and Zee, 2015; Bertolini et al., 2019).

Projections from the PHN to the VC have been demonstrated in various experimental animals (Blanks et al., 1983; Sato et al., 1983; Langer et al., 1985; Blanks, 1990; McCrea and Horn, 2006), and cholinergic neurons in the PHN have also been shown to project to various cerebellar regions, including the VC (Barmack et al., 1992a,b; Zhang et al., 2014; Sugimura and Saito, 2021). In contrast to the PHN-VC pathway, only one report—conducted in cats—has identified extremely sparse projections from the INC to the VC (Røste and Dietrichs, 1988). This finding suggests that the INC does not significantly interact with the VC; however, lesions within the VC have been shown to affect vertical gaze holding (Zee et al., 1981). Therefore, in this study, we aimed to obtain anatomical evidence of an INC-VC pathway via rabies virus-based transsynaptic tracing.

## Materials and Methods

All experimental procedures were approved by the Animal Care Committee of Nara Medical University and the Experimental Animal Committee of the National Institute for Physiological Sciences. The experiments were carried out in accordance with the guidelines outlined by the US National Institutes of Health regarding the care and use of animals for experimental research. A total of 15 male Long–Evans rats were used in this study. Tracer and AAV injections were performed in rats aged 7–8 postnatal weeks.

### Tracer injection

The tracer injection methods used were similar to those described previously (Sugimura and Saito, 2021). Dextran-conjugated Alexa Fluor 488 (5% in Tris-buffered saline, Invitrogen/Thermo Fisher Scientific) was injected into the FL, uvula and nodulus (UN), lobules I–II in the vermis, and crus I–II. The tracers were injected unilaterally into the right FL and crus I–II and into the midline regions of the UN and lobules I–II in the vermis. Under inhalation anesthesia with 2–3% isoflurane, the rats were placed in a stereotaxic apparatus. Using a dental drill, a small hole was drilled in the cranium over the parafloccular lobe, the midline region of the posterior vermis, the midline region of the anterior vermis, or the posterior hemisphere for tracer injection into the FL, UN, lobules I–II, or crus I–II, respectively. A glass micropipette with a tip diameter of 40–60 μm was connected to a Hamilton syringe with polyethylene tubing and filled with oil. Dextran-conjugated Alexa Fluor 488 was drawn into the tip of the micropipette. For injection into the FL, the tip of the micropipette was introduced horizontally, 20° posterior to the mediolateral axis, and inserted 2.7 mm beneath the surface to pass through the parafloccular lobe and enter the FL. For the injections into the other three regions, the tip was positioned on the midline, 4.8 mm posterior to the lambda, at a depth of 4.8 mm for the UN; on the midline, 1.0 mm posterior to the lambda, at a depth of 4.8 mm for lobules I–II in the vermis; and 4.5 mm lateral to the midline, 5.0 mm posterior to the lambda, at a depth of 1.2 mm for crus I–II. Tracer injection was performed by applying pressure for >5 min per site. The tracer was injected at two sites in the FL and at one site in the other region. After the injection of a total of 0.8–1.0 μl of tracer per rat, the pipette was maintained at its position for 5 min before withdrawal. Following incision suturing, an antibiotic (gentamicin ointment; MSD K.K.) was applied to the wound, and a nonsteroidal anti-inflammatory drug (flunixin meglumine, 1.5 mg/kg; Sigma-Aldrich) was applied subcutaneously.

### Virus preparation and virus injection for transsynaptic tracing

The glycoprotein (G)-deleted rabies virus pseudotyped with avian sarcoma leucosis virus envelope protein (EnvA), EnvA-HEP-ΔG-mCherry, was produced as previously described (Osakada and Callaway, 2013; Mori and Morimoto, 2014) with minor modifications. First, to obtain the G-deleted rabies virus HEP strain, BHK-T7/9 cells were transfected with plasmids encoding the entire rabies virus genome with mCherry instead of rabies glycoprotein (pcDNA-HEP-ΔG-mCherry) and helper plasmids encoding the rabies nucleoprotein (RN; pcDNA-RN), phosphoprotein (RP; pcDNA-RP), and polymerase (RL; pcDNA-RL) genes using TransIT-LT1 reagent (Takara Bio), as described in the manufacturer's instructions. Once the mCherry fluorescence signal increased, the culture medium containing the rabies virus HEP-ΔG-mCherry was collected and transferred to the BHK-21 cell line expressing the SADcvsG glycoprotein to increase the titer and amount of HEP-ΔG-mCherry virus. Second, the BHK-21 cell line expressing EnvA was infected with the collected HEP-ΔG-mCherry virus. Because HEP-ΔG-mCherry lacks glycoprotein in the genome, the culture media contained the pseudotyped rabies virus EnvA-HEP-ΔG-mCherry. The virus was concentrated via centrifugation of the culture medium. We obtained the EnvA-HEP-ΔG-mCherry (referred to in the text as EnvA-RVdG-RFP) virus with an infectious titer of 2.3 × 10^8^ IU/ml in HEK293-TVA (tumor virus receptor A) cells. Retrograde AAVs were used to express rabies G and TVA for rabies virus tracing in an axonal projection area-specific manner. The sequence between the two loxP sites of the FLEX plasmids, pAAV-CA-FLEX-SADcvsG and pAAV-EF1α-FLEX-GT (Addgene plasmid 26198), was inverted by Cre recombinase (M0298, New England Biolabs) in vitro. The modified plasmids pAAV-CA-SADcvsG and pAAV-EF1α-GT were used to produce retrograde AAV vectors (AAV2retro-CA-SADcvsG and AAV2retro-EF1α-GFP-TVA). The titers of AAV2retro-CA-SADcvsG (referred to in the text as AAV2retro-G) and AAV2retro-EF1α-GFP-TVA (referred to in the text as AAV2retro-GFP-TVA) were 2.0 × 10^12^ genomic copies (GC)/ml and 9.8 × 10^11^ GC/ml, respectively. In this study, AAV2retro-G and AAV2retro-GFP-TVA are referred to as helper AAV2retro viruses.

For transsynaptic labeling, a total of 1.0 μl of a 1:1 mixture of AAV2retro-GFP-TVA and AAV2retro-G was injected into the right FL (0.5 μl into each of the two sites) or into the midline region of the UN (1.0 μl into a single site). Three weeks later, 0.8 μl of EnvA-RVdG-RFP was injected unilaterally into the right (Figs. 3–5) or left (Fig. 6) PHN and medial vestibular nucleus (MVN). Injection into only the PHN and MVN was considered difficult because the two nuclei are located adjacent to each other; therefore, the virus was injected into both the PHN and MVN. The tip was positioned 0.5–1.0 mm lateral to the midline, 3.8 mm posterior to the lambda, at a depth of 6.5 mm from the surface of the cerebellum for injection into the PHN and MVN. The virus was pressure injected via the same procedure used for the tracer injection. Five days after the injection, the cells were maintained to allow infection with EnvA-RVdG-RFP, transsynaptic spread of the virus, and sufficient RFP expression for labeling presynaptic neurons.

### Histological procedures

Three to five days after tracer injection or 5 d after rabies virus injection, the rats were anesthetized via isoflurane inhalation followed by intraperitoneal injection of a mixture of medetomidine (0.15 mg/kg), midazolam (2.0 mg/kg), or butorphanol (2.5 mg/kg). The rats were then transcardially perfused with 0.01 M phosphate-buffered saline (PBS), pH 7.4, followed by 4% paraformaldehyde in 0.1 M phosphate buffer. The brain was removed and postfixed in the same fixative for 1 d. The injection sites and the brain areas were cut frontally into 60 μm sections via a microslicer (Dosaka EM). For the tracer experiments, some slices were counterstained with red fluorescent Nissl stain (1:200, NeuroTrace 530/615; Thermo Fisher Scientific). For transsynaptic tracing, to enhance GFP signals, the sections were incubated overnight at room temperature with chicken IgY anti-GFP antibody (1:1,000; ab13970, Abcam) in PBS containing 5% bovine serum albumin, 0.1% Triton X-100, and 0.1% NaN_3_, followed by Alexa Fluor 488 goat anti-chicken IgY (1:400; ab150169, Abcam) in the same solution at room temperature for 2 h. The sections were mounted on MAS-coated slides (Matsunami Glass) using antifade medium (ProLong Gold Antifade Reagent, Invitrogen) or a DAPI-containing fluoromount (DAPI Fluoromount-G, Southern Biotech, 0100-20).

### Observation and data analysis

Images of the brain sections were captured under a laser-scanning confocal microscope (C2+, Nikon) or a fluorescence microscope (BZ-X710, Keyence). The images were then processed and analyzed via Fiji (Schindelin et al., 2012). For the tracer experiments of the INC, every other 60 µm coronal section from the bregma −5.04 to −6.84 mm was investigated. For transsynaptic tracing, every other 60 µm coronal section was investigated from the bregma −10.32 to −12.84 mm for the PHN, from the bregma −9.96 to −13.08 mm for the MVN, and from the bregma −5.04 to −6.84 mm for the INC; all coordinates were determined according to the rat brain atlas (Paxinos and Watson, 2018). To determine whether the number of RFP-expressing neurons demonstrated a rostrocaudal gradient within the INC, we separated the INC into rostral, intermediate, and caudal parts. Each of the parts, consisting of five alternating sections, was 600 μm in length in the rostrocaudal direction. GFP- or RFP-expressing neurons were manually counted via the cell counter plugin in Fiji. The detection threshold for GFP- or RFP-labeled neurons was arbitrarily defined. Neurons labeled with both GFP and RFP were selected from neurons that showed positive fluorescence in both the RFP and the GFP fluorescence images. For transsynaptic tracing, cell counts were performed for the PHN and MVN ipsilateral to the site of rabies virus injection to identify starter neurons that expressed both GFP and RFP and in the bilateral INC to identify RFP-expressing neurons. All values are shown as the means ± SDs, and the error bars in the figures represent the SDs. Unless otherwise noted, the number (*n*) refers to the number of rats analyzed. Statistical analysis was performed via the Wilcoxon signed-rank test or repeated-measure one–way ANOVA followed by the Tukey–Kramer test for multiple comparisons, for which an adjusted *p* value was calculated. The normality of the data was determined via the Shapiro–Wilk test. Differences in variance among the groups were assessed via the Brown–Forsythe test. The analyses were performed via GraphPad Prism 8 (GraphPad Software). The threshold for statistical significance was defined as *p* < 0.05.

## Results

### INC neurons do not project directly to the VC

**Figure 1.**
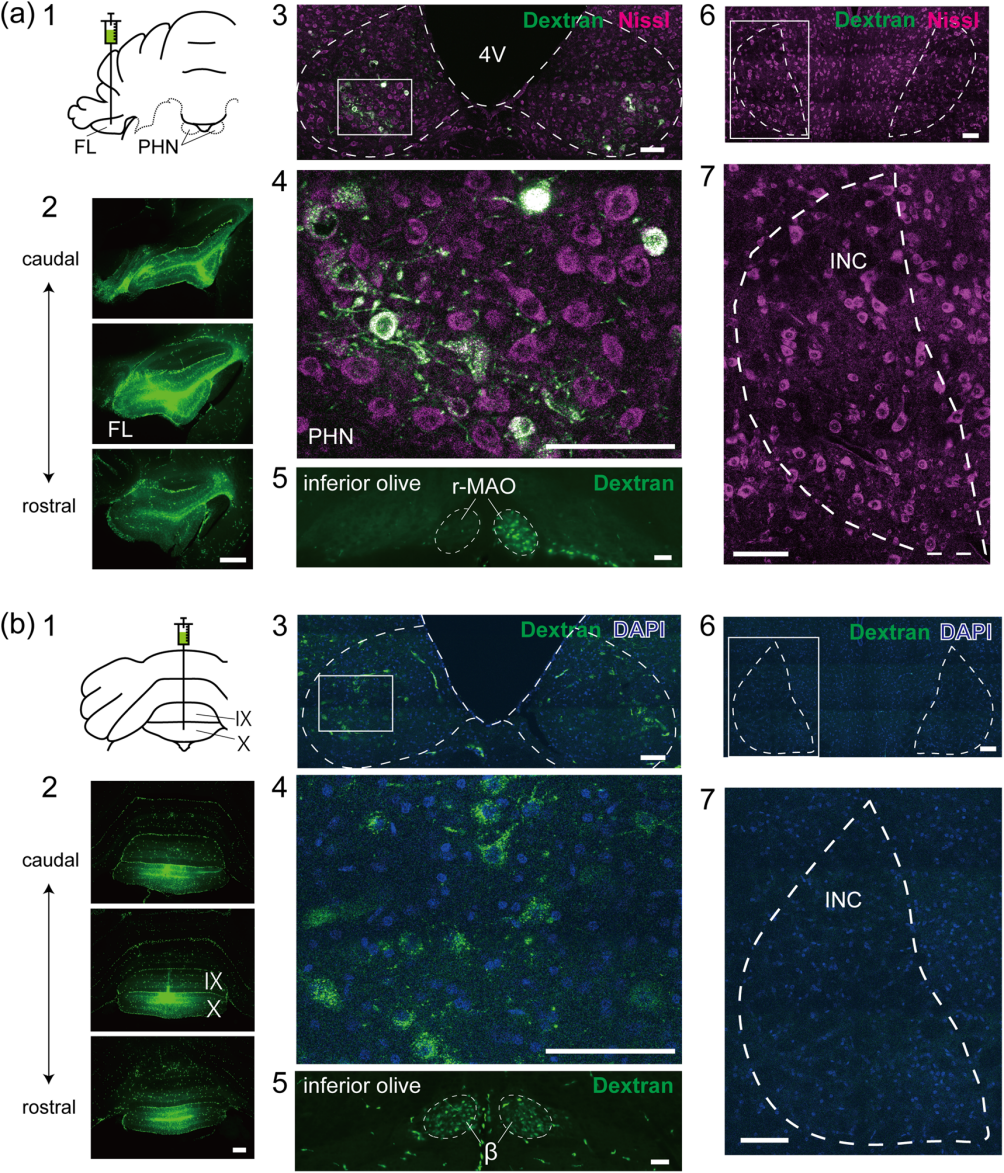
Neurons retrogradely labeled via tracer injection into the VC. ***a*1**, ***b*1**, Schematic drawings of the injection of dextran-conjugated Alexa Fluor 488 into the FL (***a*1**) and UN (***b*1**) of rats. ***a*2**, ***b*2**, Fluorescence photomicrographs of the injection sites. Each photo is separated by 300 μm in the rostrocaudal direction. ***a*3**, ***b*3**, Confocal images of frontal sections that include the PHN. The dashed line approximately indicates the boundary of the PHN. ***a*4**, ***b*4**, Enlarged images of the box in **3**. ***a*5**, ***b*5**, Fluorescence photomicrographs of frontal sections that include the inferior olive. ***a*6**, ***b*6**, Confocal images of frontal sections that include the INC. The dashed line approximately indicates the boundary of the INC. ***a*7**, ***b*7**, Enlarged images of the box in **6**. Scale bars: ***a*2**, ***b*2**, 500 μm; ***a*3–7**, ***b*3–7**, 100 μm. 4 V, fourth ventricle; FL, flocculus; UN, uvula and nodulus; r-MAO, rostral part of the medial accessory olive; beta, subnucleus beta of the inferior olive. The Roman numerals indicate the cerebellar lobules.

To investigate the INC-VC pathway, we injected dextran-conjugated Alexa Fluor 488 into two distinct regions of the VC, the FL (*n* = 2) and lobules IX and X of the cerebellar vermis (UN; *n* = 2; Fig. 1*a*1,*b*1). The tracers were widely distributed both mediolaterally and rostrocaudally within the FL and UN (Fig. 1*a*2,*b*2). Although retrogradely labeled neurons were observed in the PHN (Fig. 1*a*3,4,*b*3,4), no labeled neurons were observed in the INC (Fig. 1*a*6,7,*b*6,7). Successful tracer injection was confirmed by observing retrogradely labeled neurons in the rostral part of the medial accessory olive (r-MAO) contralateral to the injection site (Fig. 1*a*5) and in the subnucleus beta of the inferior olive (beta; Fig. 1*b*5), which are known to project to the FL and UN, respectively (Sugihara et al., 2004; Sugihara and Shinoda, 2004; Ando et al., 2020). These results indicate that INC neurons do not project directly to the FL or UN.

**Figure 2.**
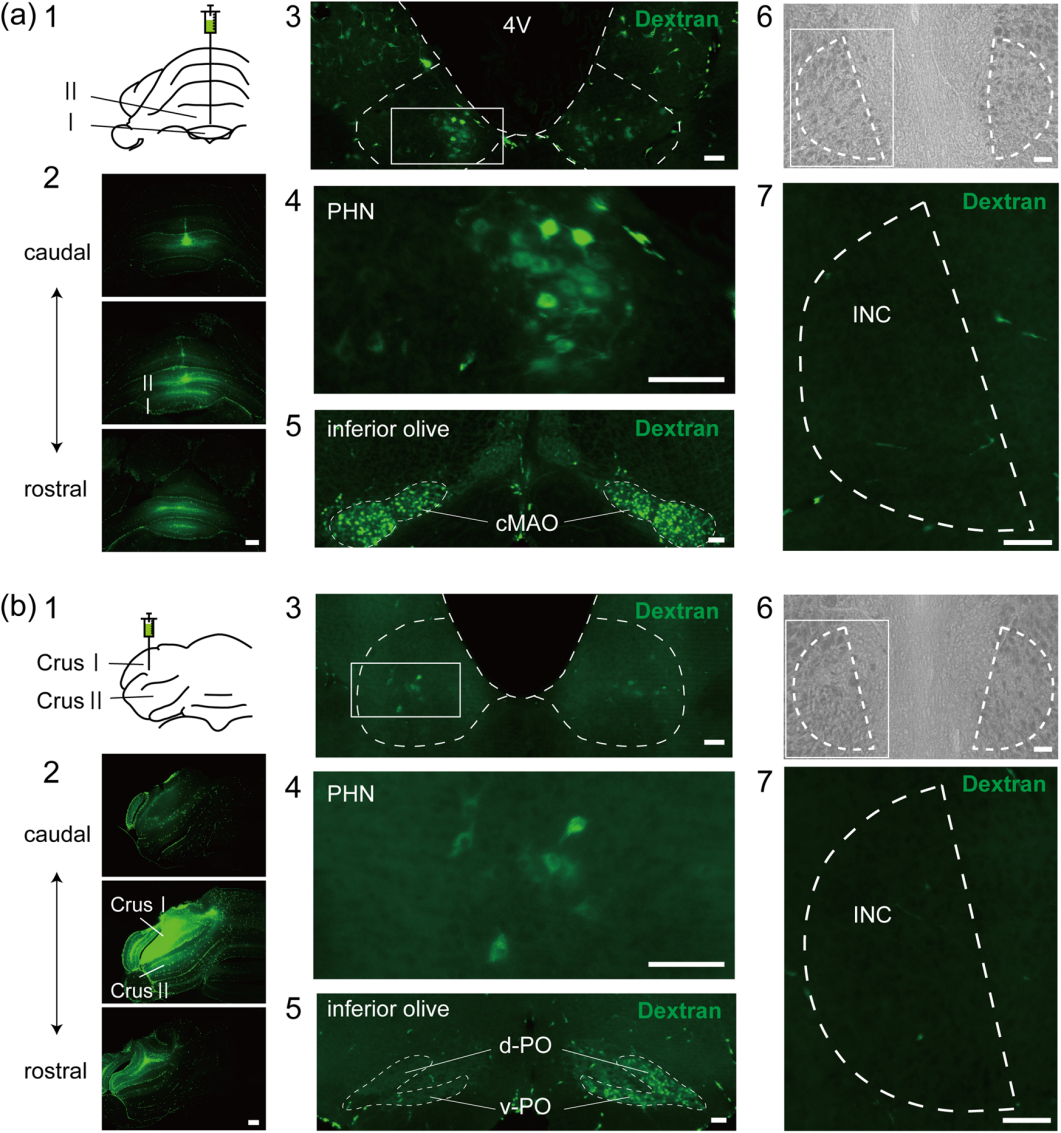
Neurons retrogradely labeled following tracer injection into lobules I–II in the vermis and crus I–II. ***a*1**, ***b*1**, Schematic drawings of the injection of dextran-conjugated Alexa Fluor 488 into lobules I–II in the vermis (***a*1**) and crus I–II (***b*1**) of rats. ***a*2**, ***b*2**, Fluorescence photomicrographs of the injection sites. Each photo is separated by 300 μm in the rostrocaudal direction. ***a*3**, ***b*3**, Fluorescence photomicrographs of frontal sections that include the PHN. The dashed line approximately indicates the boundary of the PHN. ***a*4**, ***b*4**, Enlarged images of the box in **3**. ***a*5**, ***b*5**, Fluorescence photomicrographs of frontal sections that include the inferior olive. ***a*6**, ***b*6**, Bright-field images of frontal sections that include the INC. The dashed line approximately indicates the boundary of the INC. ***a*7**, ***b*7**, Enlarged fluorescence images of the box in **6**. Scale bars: ***a*2**, ***b*2**, 500 μm; ***a*3**–**7**, ***b*3**–**7**, 100 μm. 4 V, fourth ventricle. c-MAO, caudal part of the medial accessory olive; v-PO, ventral lamella of the principal olive; d-PO, dorsal lamella of the principal olive. The Roman numerals indicate the cerebellar lobules.

Lobules I–II in the cerebellar vermis and crus I–II in the hemisphere receive vestibular inputs (Kotchabhakdi and Walberg, 1978; Matsushita and Okado, 1981; Hirai, 1983) and inputs from the PHN (Payne, 1983; Belknap and McCrea, 1988; Røste, 1989; Sugimura and Saito, 2021), respectively. Therefore, to determine the possibility that INC neurons project to these cerebellar regions rather than the VC, we injected dextran-conjugated Alexa Fluor 488 into lobules I–II in the vermis (*n* = 2; Fig. 2*a*1) and into crus I–II (*n* = 2; Fig. 2*b*1). The tracers were widely distributed both mediolaterally and rostrocaudally in both lobules I–II (Fig. 2*a*2) and crus I–II (Fig. 2*b*2). Retrogradely labeled neurons were observed in the PHN (Fig. 2*a*3,4,*b*3,4) but not in the INC (Fig. 2*a*7,*b*7). We also confirmed retrogradely labeled neurons in the caudal parts of the medial accessory olive (c-MAO), which project to lobules I–II (Sugihara and Shinoda, 2004; Fig. 2*a*5), and in the ventral lamella of the principal olive (v-PO) and the dorsal lamella of the principal olive (d-PO), which project to crus I–II (Sugihara and Shinoda, 2004) contralateral to the injection site (Fig. 2*b*5). These results indicate that INC neurons directly project neither to the VC nor to lobules I–II and crus I–II.

### Indirect pathway from the INC to the VC

**Figure 3.**
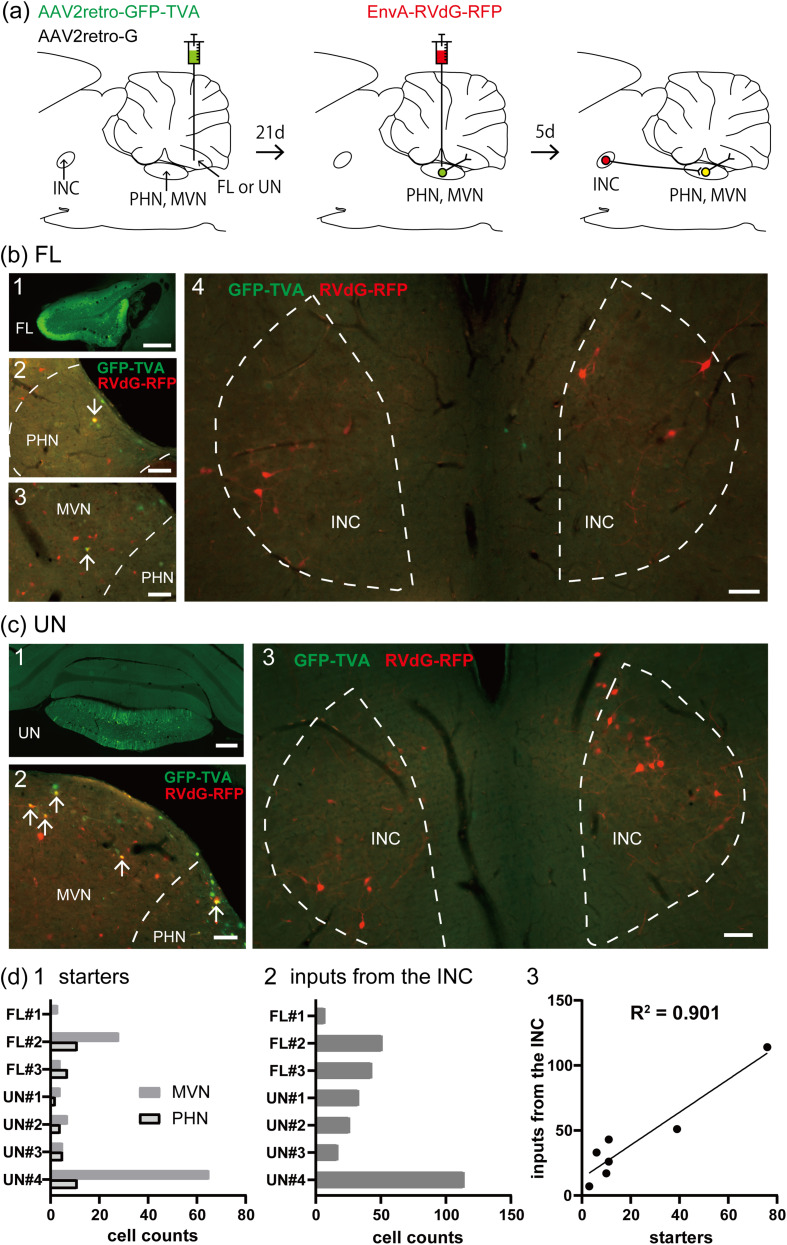
Indirect connections from the INC to the VC via the PHN and MVN. ***a***, Experimental design of retrograde monosynaptic tracing from the PHN and MVN projecting to the FL or UN of a rat. ***b*1**, ***c*1**, Fluorescence photomicrographs of GFP at the sites of injection of two helper AAV2retro viruses (AAV2retro-G and AAV2retro-GFP-TVA) on the right side of the FL (***b*1**) and the midline region of the UN (***c*1**). ***b*2,3**, ***c*2**, Example of starter cells (arrows) in the right PHN and MVN expressing both GFP and RFP. EnvA-RVdG-RFP was injected into the right side of the PHN and MVN. ***b*4**, ***c*3**, Examples of RFP-expressing neurons in the INC that received retrograde transsynaptic infection with rabies virus from starter cells. ***d*1,2**, Numbers of starter cells in the PHN and MVN (**1**) and RFP-expressing neurons in the INC (**2**), which provide inputs to the starter cells, for each rat analyzed. “FL” and “UN” represent groups where helper AAVs were injected into the FL and UN, respectively. The numbers following “FL” and “UN” are used to distinguish individual rats within each group. ***d*3**, Relationships between the number of starter cells and RFP-expressing neurons in the INC. Scale bars: ***b*1**, ***c*1**, 500 μm; ***b*2–4**, ***c*2,3**, 100 μm.

Based on the results described above and the hypothesis that the INC and the VC constitute an oculomotor neural integrator involved in the control of vertical gaze holding, the pathway from the INC to the VC may involve other areas. The PHN and the MVN receive inputs from the INC (McCrea and Baker, 1985; Belknap and McCrea, 1988; Spence and Saint-Cyr, 1988a,b; Kokkoroyannis et al., 1996) and send outputs to the VC (Zhang et al., 2014; Leigh and Zee, 2015; Sugimura and Saito, 2021). We therefore tested the possibility that the INC-FL and INC-UN pathways are mediated via the PHN and MVN via rabies virus-based transsynaptic tracing (Fig. 3*a*). Precise injections of helper adeno-associated virus 2-retro (AAV2retro) viruses into the right side of the FL (*n* = 3; Fig. 3*b*1) or the midline region of the UN (*n* = 4; Fig. 3*c*1) confirmed the presence of GFP-expressing neurons in the PHN (Fig. 3*b*2,*c*2) and the MVN (Fig. 3*b*3,*c*2). Starter cells, which were initially rabies-infected and expressed both GFP and RFP, were observed in the PHN and MVN (Fig. 3*b*2,3,*c*2), although the number of starter cells observed in the two regions differed among the rats (Fig. 3*d*1). Neurons that expressed RFP alone—those that transsynaptically received retrograde rabies virus infection via starter cells—were observed in the bilateral INCs (Fig. 3*b*4,*c*3). The number of RFP-expressing neurons in the INC was also different for each rat (Fig. 3*d*2) but was positively correlated with the number of starter cells (*R*^2 ^= 0.901; Fig. 3*d*3). INC neurons that expressed GFP alone were not observed, confirming that there were no direct projections from the INC to the FL or UN. These results indicate that the INC-VC pathway functions via synaptic connections with the PHN and MVN.

**Figure 4.**
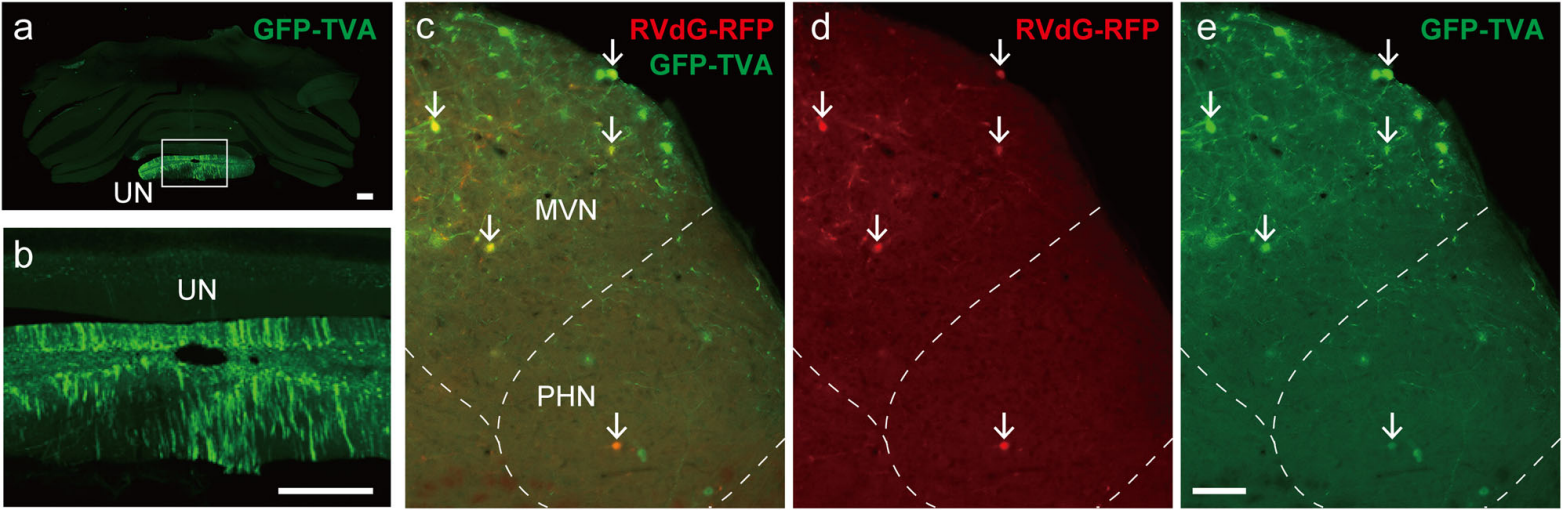
Control experiments for rabies transsynaptic tracing. ***a***, Frontal section of the cerebellum at the site of injection of AAV2retro-GFP-TVA in the UN (AAV2retro-G was not injected). ***b***, Enlarged image of the box in ***a***. ***c***, Frontal section showing the PHN and MVN after AAV2retro-GFP-TVA injection into the UN and after EnvA-RVdG-RFP injection into the PHN and MVN. The dashed line approximately indicates the boundary of the PHN and MVN. ***d***, ***e***, RFP (***d***) and GFP (***e***) images from ***c***. The arrows indicate neurons that express both GFP and RFP. Scale bars: ***a***, ***b***, 500 μm; ***e***, 100 μm.

In the PHN and MVN, neurons that exhibited RFP alone were also observed (Fig. 3*b*2,3,*c*2), which could be starter cells that express TVA at levels undetectable via GFP because of strong interactions between the wild-type TVA and EnvA-RVdG (Callaway and Luo, 2015; Kim et al., 2015). We tested this possibility by injecting helper AAV2retro without AAV2retro-G into the UN and EnvA-RVdG-RFP into the PHN and MVN to prevent transsynaptic infection (*n* = 2). All PHN and MVN neurons that expressed RFP expressed GFP (Fig. 4). These results indicate that the expression of RFP alone by PHN and MVN neurons resulted from transsynaptic infection of starter cells. These findings suggest that the neurons in the PHN and MVN that express RFP alone are interneurons that form synaptic connections and provide inputs to starter cells.

### Distribution of INC neurons that participate in the INC-VC pathway via the PHN and MVN

RFP-expressing neurons were observed in the bilateral INCs, despite the unilateral injection of rabies virus into the PHN and MVN (Fig. 3*b*4,*c*3). Therefore, the numbers of RFP-expressing neurons in the left and right INCs were compared. The number of RFP-expressing neurons did not significantly differ between the bilateral INCs when the helper AAV2 retrovirus was injected into the UN (ipsilateral, 21 ± 14; contralateral, 27 ± 31; *p* > 0.999; *n* = 4), nor did it significantly differ between the ipsilateral (13 ± 10) and contralateral (21 ± 15) sides when the virus was unilaterally injected into the FL (*p* = 0.250; *n* = 3). In addition to comparing the absolute neuron numbers, we calculated the bias index for each animal to assess laterality via the formula (ipsi − contra) / (ipsi + contra), where “ipsi” and “contra” refer to the number of RFP-expressing neurons in the ipsilateral and contralateral INCs, respectively. The bias index values were −0.020 ± 0.212 for the UN group (Fig. 3*c*; *n* = 4) and −0.275 ± 0.220 for the FL group (Fig. 3*b*; *n* = 3). One-sample *t* tests indicated that the mean bias indices in both the UN and FL groups did not significantly differ from zero (*p* = 0.864 and *p* = 0.163, respectively). These results suggest that the INC neurons that received transsynaptic infection from starter cells did not show marked laterality.

**Figure 5.**
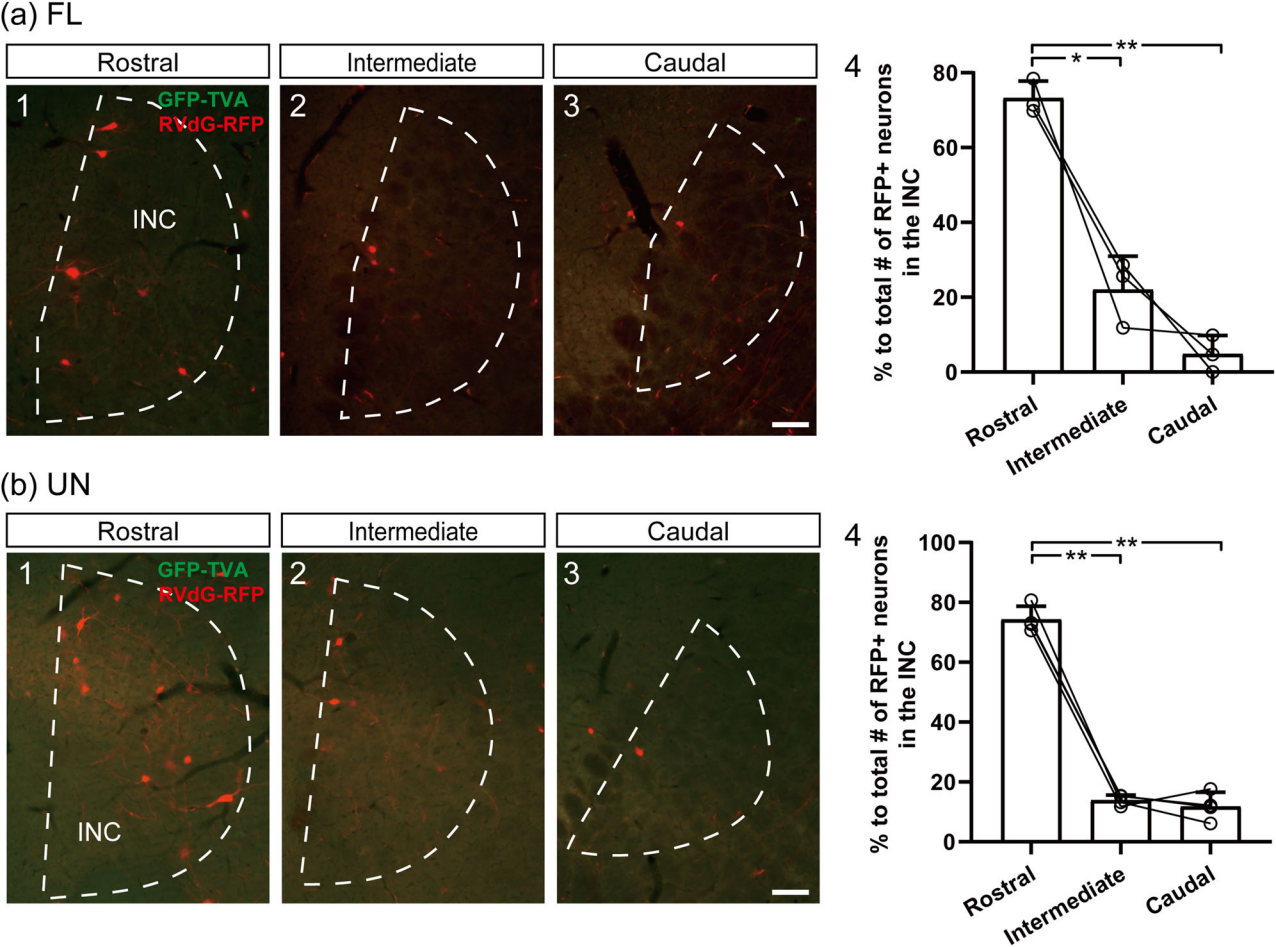
Distribution of RFP-expressing neurons in the rostral–caudal regions of the INC. ***a*1–3**, ***b*1–3**, Fluorescence photomicrographs of frontal sections that include the INC after rabies virus transsynaptic tracing (related to Fig. 3). RFP-expressing neurons in the rostral (**1**), intermediate (**2**), and caudal (**3**) regions of the INC following injection of the two helper AAV2 retroviral vectors into the FL (***a***) and UN (***b***). (***a*4**, ***b*4**) Comparison of the percentages of RFP-expressing (RFP+) neurons in each region with respect to the total number of RFP-expressing neurons in the INC (***a*4**, *n* = 3; ***b*4**, *n* = 4). Individual plots indicate data obtained from individual rats. The error bars represent the SDs. Scale bars: ***a*3**, ***b*3,** 100 μm. Asterisks indicate a significant difference between groups (*p < 0.05; **p < 0.01).

We next examined the rostrocaudal distribution of RFP-expressing neurons in the INC. RFP-expressing neurons were more frequently observed in the rostral region than in the intermediate and caudal regions following injection into both the FL (Fig. 5*a*1–3) and UN (Fig. 5*b*1–3). Comparisons of RFP-expressing neurons in the three regions relative to the total number of RFP-expressing neurons in the INC revealed that the percentage in the rostral region was significantly greater than that in the intermediate and caudal regions following injection into both the FL (*p* = 0.0398 and *p* = 0.0002, respectively; *n* = 3; Fig. 5*a*4) and UN (*p* = 0.0003 and *p* = 0.0017, respectively; *n* = 4; Fig. 5*b*4). These results indicate that the distribution of RFP-expressing neurons depends on the rostrocaudal location within the INC.

### The INC is connected to the FL via the bilateral PHNs and MVNs

**Figure 6.**
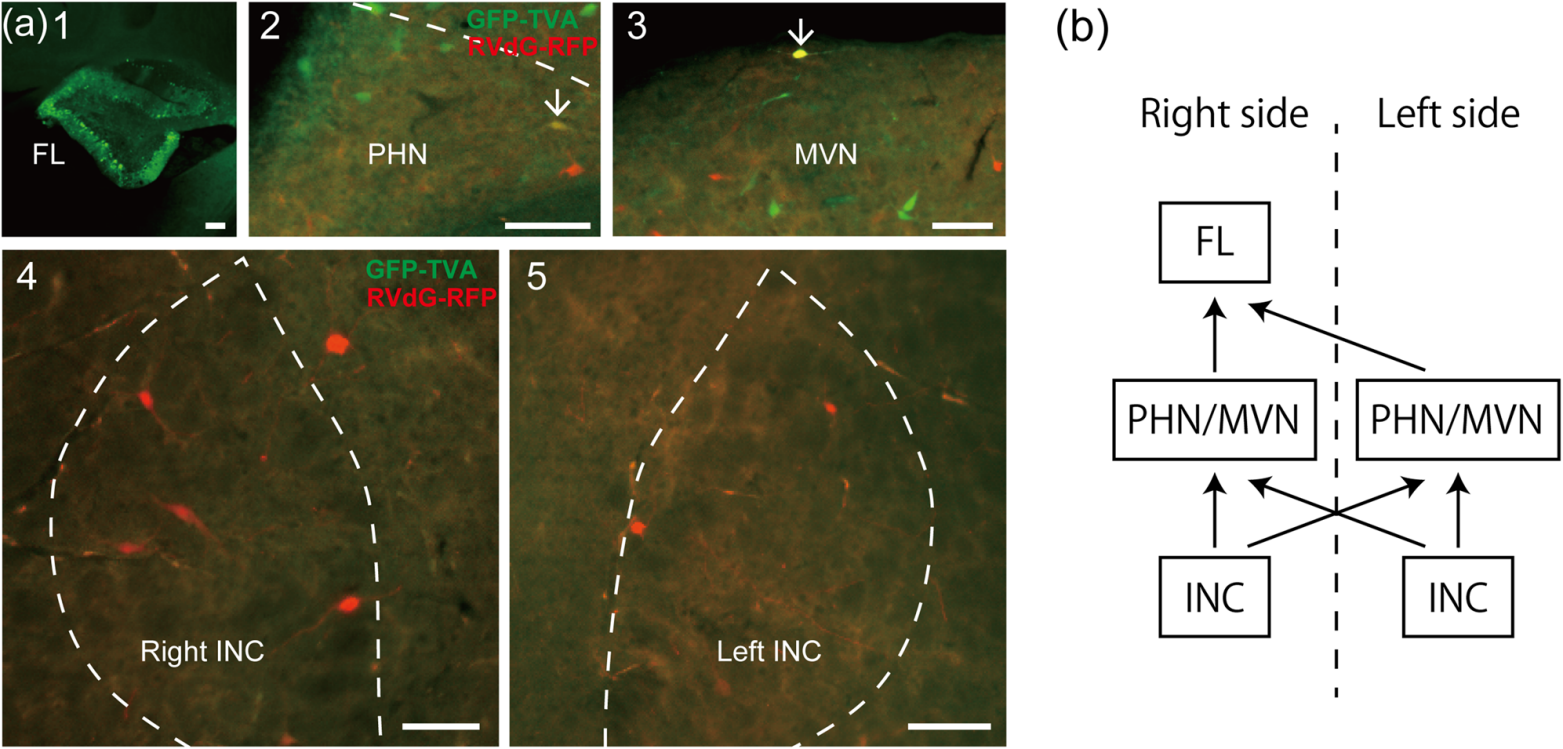
The indirect INC-FL pathway is mediated via the bilateral PHNs and MVNs. ***a*1**, Fluorescence photomicrograph of GFP at the site of injection of the two helper AAV2retro viruses (AAV2retro-G and AAV2retro-GFP-TVA) on the right of the FL. ***a*2,3**, Example starter cells (arrows) expressing both GFP and RFP in the left PHN and MVN. EnvA-RVdG-RFP was injected into the left PHN and MVN. Figure 3 shows that the rabies virus was injected into the right PHN and MVN. ***a*4,5**, Fluorescence photomicrographs of frontal sections that include the INC. RFP-expressing neurons, which received retrograde transsynaptic infection with rabies virus from starter cells, were observed in the right (**4**) and left INC (**5**). ***b***, Schematic diagram of the pathway from the INC to the FL via the bilateral PHNs and MVNs revealed in this study. The diagram shows the neural pathway to the right FL only. Scale bars: ***a*1**, 200 μm; ***a*2–5**, 100 μm.

Finally, we investigated whether INC neurons connect to the FL via the unilateral or bilateral PHNs and MVNs. Following AAV2retro-G and AAV2retro-GFP-TVA injections into the right FL (Fig. 6*a*1) and EnvA-RVdG-RFP injections into the left PHN and MVN in three rats, starter cells were observed in the left PHN and MVN, and RFP-expressing neurons were observed in the bilateral INCs (Fig. 6*a*2–5). Together with the preceding results (Fig. 3), these results indicate that INC neurons connect to the FL via the bilateral PHNs and MVNs. Figure 6*b* summarizes the pathway from the INC to the FL mediated via the PHN and MVN on the basis of our results on retrograde labeled neurons.

## Discussion

In this study, we aimed to obtain anatomical evidence of an INC-VC pathway. We did not find that INC neurons directly projected to the FL or UN via retrograde tracing experiments. However, rabies virus-based transsynaptic tracing experiments revealed that INC neurons received retrograde transsynaptic infection with the virus via projection neurons to the FL and UN in the PHN and MVN. These results indicate that the neural pathway from the INC to the VC is formed via synaptic connections with the PHN and MVN.

**Figure 7.**
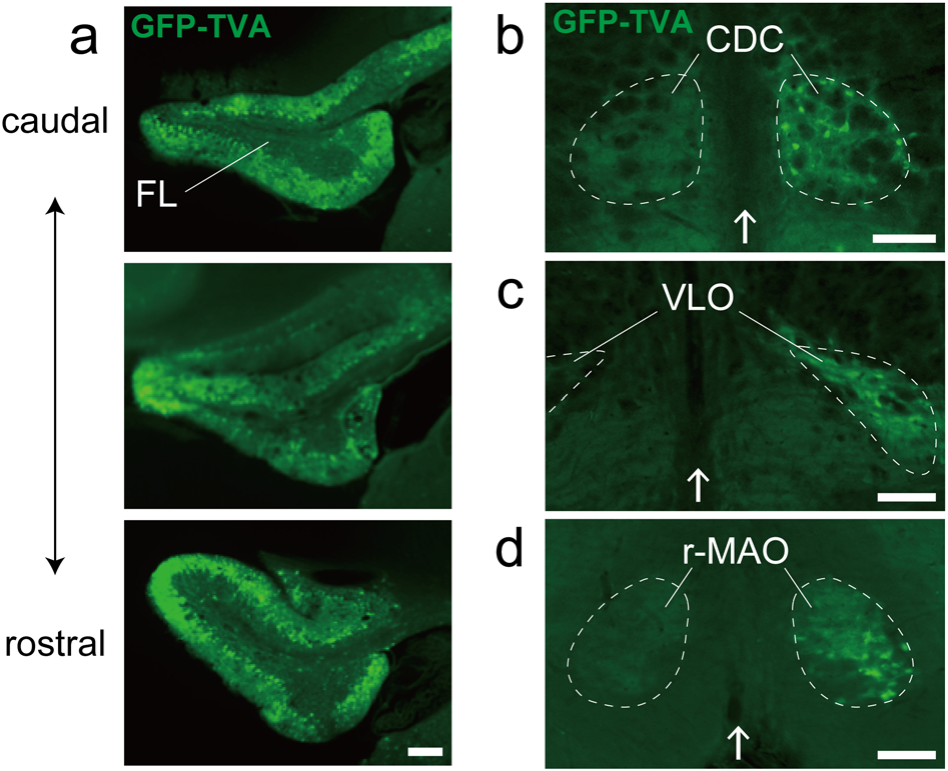
Inferior olive neurons retrogradely labeled following helper AAV2 retrovirus injection into the FL. ***a***, Fluorescence photomicrographs at the sites of injection of the two helper AAV2 retroviral vectors (AAV2retro-GFP-TVA and AAV2retro-G) in the right FL (related to Fig. 3). Each photo is separated by 300 μm in the rostrocaudal direction. ***b–d***, Fluorescence photomicrographs of frontal sections that include the CDC and VLO of the inferior olive and the r-MAO. The arrows indicate the midline. Scale bars: ***a***, 200 μm; ***b–d***, 100 μm. FL, flocculus; CDC, caudal dorsal cap; VLO, ventrolateral outgrowth; r-MAO, rostral part of the medial accessory olive.

When dextran-conjugated Alexa Fluor 488 or AAV2retro-GFP-TVA was injected into the FL or UN of the rats, no retrogradely labeled neurons were observed in the INC, although many labeled neurons were observed in the PHN (Figs. 1, 3). The cerebellum is composed of a zonal structure with specific input–output relationships as its functional unit. Local stimulation of distinct zones (the H-zone and V-zone) in the FL has been shown to induce horizontal and vertical eye movements in various animals, including rabbits (Dufossé et al., 1977; Nagao et al., 1985), cats (Sato and Kawasaki, 1984, 1990), and monkeys (Balaban and Watanabe, 1984). Further detailed studies in mice and rabbits based on the complex spike modulation of floccular Purkinje cells in response to optokinetic stimulation revealed that the cells in Zones 1 and 3 (HA zones) preferentially respond to rotation about the horizontal axis oriented at the 135° ipsilateral azimuth, whereas those in Zones 2 and 4 (VA zones) preferentially respond to rotation about the vertical axis (De Zeeuw et al., 1994; Schonewille et al., 2006). Climbing fiber inputs to Purkinje cells in the HA and VA zones originate mainly from neurons in the ventrolateral outgrowth (VLO) and the caudal dorsal cap (CDC) of the contralateral inferior olive, respectively (Tan et al., 1995; Sugihara et al., 2004; Voogd and Wylie, 2004; Schonewille et al., 2006). In the present study, we observed retrogradely GFP-labeled neurons in the contralateral VLO and CDC following injection of helper AAV2 retroinjected into the FL (Fig. 7). These findings indicate that the AAV viruses were injected into both the HA and VA zones in the FL and that the absence of retrogradely GFP-labeled neurons in the INC was not due to a failed AAV injection into the HA zones.

Our transsynaptic tracing experiments using the rabies virus revealed that the INC is connected to both the FL and UN via the PHN and MVN. Although these indirect INC-VC pathways pass through the region of the horizontal integrator, they may play a role in vertical gaze holding. Indeed, previous studies have shown that postaccadic drift appears to be induced during vertical eye movements and that horizontal gaze holding is impaired when the PHN and MVN are chemically lesioned or pharmacologically inactivated (Cannon and Robinson, 1987; Kaneko, 1997). The induction of vertical drifts despite the impairment of horizontal integrators may be due to insufficient functioning of the INC-VC pathway via the PHN and MVN. Extracellular unit spike recording experiments have revealed that some PHN and MVN neurons exhibit tonic activity related to vertical eye position (Baker et al., 1976; Tomlinson and Robinson, 1984), suggesting that these neurons are involved in the indirect INC-VC pathway.

Our study further clarified the characteristics of the INC-VC pathway. First, INC neurons that participate in the INC-VC pathway are present on both sides of the INC and exhibit no significant laterality (Fig. 3). Second, the INC neurons involved in the INC-VC pathway are located mainly in the rostral part of the INC (Fig. 5). A previous study on vertical eye movements showed that unilateral injections of a retrograde tracer into the entire vestibular complex result in retrogradely labeled cells in both the ipsilateral and contralateral INCs, largely in their rostral regions (Spence and Saint-Cyr, 1988b). These distributions of INC neurons are similar to our results; therefore, the pathway from the INC to the vestibular nuclei may be common in vertical eye movements and vertical gaze holding. Third, the pathway from the INC to the FL is mediated via both the ipsilateral and contralateral PHNs and MVNs (Fig. 6). In our study, virus-infected neurons in the bilateral INCs were observed following unilateral injection of the AAV virus into the FL, suggesting that the pathway from the INC reached the bilateral FLs via the bilateral PHNs and MVNs. Previous studies have shown that postsaccadic drifts in vertical eye movements are less pronounced in subjects with unilateral PHN and MVN lesions than in those with bilateral lesions (Cannon and Robinson, 1987; Kaneko, 1997). The weaker symptomology experienced in subjects with unilateral lesions of the PHN and MVN may be due to incomplete disruption of the INC-FL pathway, which is mediated via the bilateral PHNs and MVNs.

Cell groups in paramedian tracts (PMTs), which are scattered along the paramedian regions of the pons and medulla, project to the floccular region (Büttner-Ennever et al., 1989; Büttner-Ennever and Horn, 1996). All premotor neurons that project to ocular motoneurons send axon collaterals to the PMT (Büttner-Ennever et al., 1989; Büttner-Ennever and Horn, 1996). A previous monkey study showed that the INC projects to the PMT (Büttner-Ennever and Horn, 1996). Lesions within or inactivation of PMT cell groups lead to downbeat nystagmus (Nakamagoe, 2000; Nakamagoe et al., 2012; Helmchen et al., 2013), upbeat nystagmus (Tian et al., 2023), and omnidirectional gaze-evoked nystagmus (Anagnostou et al., 2009). These findings suggest that the pathway from the INC to the FL via the PMT is involved in vertical gaze holding. Because some PMT neurons encode activity similar to that of oculomotor neurons, PMT cell groups may send an efferent copy of the motor command to the FL (Cheron et al., 1995; Horn and Leigh, 2011; Leigh and Zee, 2015). The efference copy is used for purposes such as motion prediction rather than for the direct control of eye position. Therefore, the INC-FL pathway via the PMT may not substantially participate in gaze holding. Previous anatomical methods did not clarify how the INC connects to the VC; therefore, the neural networks around the VC in the vertical oculomotor integrator remain unclear. Our present study, which uses the rabies virus-based transsynaptic tracing method, demonstrated that the INC communicates with the VC via the PHN and the MVN. This finding provides clues as to how the INC interacts with the VC and helps gain insights into the network mechanisms responsible for vertical gaze holding.

## Synthesis

Reviewing Editor: David Schoppik, New York University Grossman School of Medicine Decisions are customarily a result of the Reviewing Editor and the peer reviewers coming together and discussing their recommendations until a consensus is reached. When revisions are invited, a fact-based synthesis statement explaining their decision and outlining what is needed to prepare a revision will be listed below. The following reviewer(s) agreed to reveal their identity: NONE. Note: If this manuscript was transferred from JNeurosci and a decision was made to accept the manuscript without peer review, a brief statement to this effect will instead be what is listed below.

The revisions strengthen the framing and data in the manuscript and adequately address all of the Reviewers' concerns.
